# RELATIVE AEROBIC LOAD OF WALKING IN PEOPLE WITH MULTIPLE SCLEROSIS

**DOI:** 10.2340/jrm.v56.13352

**Published:** 2024-02-14

**Authors:** Arianne S. GRAVESTEIJN, Sjoerd T. TIMMERMANS, Jip AARTS, Hanneke E. HULST, Brigit A. DE JONG, Heleen BECKERMAN, Vincent DE GROOT

**Affiliations:** 1 MS Center Amsterdam, Rehabilitation Medicine, Vrije Universiteit Amsterdam, Amsterdam UMC location VUmc; 2 Amsterdam Movement Sciences Research Institute, Rehabilitation & Development; 3 Amsterdam Neuroscience Research Institute, Neuroinfection & Neuroinflammation, Amsterdam; 4 Leiden University, Faculty of Social Sciences, Institute of Psychology, Health, Medical and Neuropsychology Unit, Leiden; 5 Department of Human Movement Sciences, Faculty of Behavioral and Movement Sciences, Vrije Universiteit Amsterdam, Amsterdam Movement Sciences; 6 MS Center Amsterdam, Anatomy and Neuroscience, Vrije Universiteit Amsterdam, Amsterdam UMC location VUmc; 7 MS Center Amsterdam, Neurology, Vrije Universiteit Amsterdam, Amsterdam UMC location VUmc, Amsterdam, The Netherlands

**Keywords:** anaerobic threshold, cardiorespiratory fitness, energy metabolism, gait, rehabilitation

## Abstract

**Objective:**

To examine the energy demand of walking relative to aerobic capacity in people with multiple sclerosis.

**Design:**

Cross-sectional cohort study.

**Patients:**

A total of 45 people with multiple sclerosis (32 females), median disease duration 15 years (interquartile range (IQR) 9; 20), median Expanded Disability Status Scale 4 (min–max range: 2.0; 6.0).

**Methods:**

Aerobic capacity, derived from a cardiopulmonary exercise test and gas exchange measurements, assessed during a 6-min overground walk test at comfortable speed, were analysed. The relative aerobic load of walking was determined as the energy demand of walking relative to oxygen uptake at peak and at the first ventilatory threshold. Healthy reference data were used for clinical inference.

**Results:**

People with multiple sclerosis walk at a mean relative aerobic load of 60.0% (standard deviation 12.8%) relative to peak aerobic capacity, and 89.1% (standard deviation 19.9%) relative to the first ventilatory threshold. Fourteen participants walked above the first ventilatory threshold (31%). Peak aerobic capacity was reduced in 45% of participants, and energy demands were increased in 52% of participants.

**Conclusion:**

People with multiple sclerosis walk at a relative aerobic load close to their first ventilatory threshold. A high relative aerobic load can guide clinicians to improve aerobic capacity or reduce the energy demands of walking.

LAY ABSTRACT

People living with multiple sclerosis often experience walking problems, which may be increased by a high energy demand during walking and/or reduced aerobic fitness. In 45 people with multiple sclerosis, this study examined how much of the subject’s total available energy is required during walking; the so-called relative aerobic load of walking. Compared with healthy subjects, those with multiple sclerosis were found to use more energy during walking, while having less energy available. This may result in early exhaustion during walking and can have a negative effect on the daily lives of people with MS. It may be possible to address these effects by appropriate treatment; for example, through aerobic exercise training to improve aerobic fitness or the use of assistive devices to reduce energy demands during walking.

In multiple sclerosis (MS), inflammation and degeneration of the central nervous system leads to numerous symptoms, such as loss of muscle strength, poor balance control, pain and fatigue, which, in turn, impact on daily activities, such as walking (1). In the first year of the disease approximately one-quarter of people living with MS (pwMS) already report problems with walking (2), increasing to almost half within 5 years after diagnosis (2). Walking problems consist of spatio-temporal changes (e.g. reduced walking speed, smaller step length, and increased double-support phase), which can contribute to loss of balance control and increased fall risk (3). Walking is considered one of the most important activities of daily life, since it is essential for daily functioning and is closely linked to quality of life (4).

The increased energy demands of walking, together with reduced aerobic capacity, may enhance MS-related walking problems. Several symptoms typical of MS, such as loss of muscle strength and balance control, can contribute to increased energy demands of walking. When walking at speeds similar to healthy controls (HC), pwMS require more energy (5). Furthermore, a systematic review of 40 studies showed that pwMS have a significantly reduced peak aerobic capacity, or so-called cardiorespiratory fitness (V̇O_2peak_), compared with age- and sex-matched HC (V̇O_2peak_ 25.5 mL/kg/min in pwMS vs V̇O_2peak_ 30.9 mL/kg/min in HC) (6). Moreover, increased energy demands may result in fatigue, which can lead to a more sedentary lifestyle that further reduces peak aerobic capacity, resulting in a downward spiral (7).

In addition to peak aerobic capacity, another important threshold can be determined during a cardiopulmonary exercise test (CPET): the first ventilatory threshold (VT1). The VT1 is the threshold between mild and moderate exercise intensity, and is the point at which aerobic energy supply is supplemented by anaerobic energy to sustain the required energy demand (8). Activities above the VT1 can be performed for a longer period of time, but will eventually lead to exhaustion, whereas activities below VT1 can be sustained for very long periods. A secondary ventilatory threshold (VT2), the threshold between moderate and high exercise intensity, can also be determined during a CPET (8). Activities performed above VT2 can be sustained only for very brief periods.

The energy demand of walking can be expressed both relative to the peak aerobic capacity (%V̇O_2peak_) and relative to VT1 (%V̇O_2VT1_). Walking above a relative aerobic load of 100%V̇O_2VT1_ will result in exhaustion, affecting walking ability (9). Currently no normative values exist, but relative aerobic load has been shown to range between 36% and 49% V̇O_2peak_ in HC study populations (10, 11).

The relative aerobic load of walking is an, as yet, unexplored measure of physiological strain of walking in pwMS, which can give a better insight into the underlying limiting factors by combining the energetic demand of walking with the aerobic capacity. Therefore, the objective of the current study was to examine the relative aerobic load of walking (%V̇O_2peak_, %V̇O_2VT1_) in pwMS who experience walking problems. Both aerobic capacity and the energy demands of walking differ between females and males. In general, males with MS have a higher aerobic capacity, but also use more energy during walking compared with females (6, 12). It is therefore hypothesized that the relative aerobic load of walking would be similar for females and males. As regards overall relative aerobic load, it is hypothesized that a reduced peak aerobic capacity and an increased energy demand of walking will cause pwMS to walk at a high relative aerobic load relative to V̇O_2peak_, and close to 100%V̇O_2VT1_.

## METHODS

### Study design

A cross-sectional analysis of physiological and walking parameters was performed in 2 prospective cohorts (Exercise PRO-MS study and Clinical Care Protocol for Gait Disorders in MS) with similar testing procedures and analyses. This manuscript presents the first data collected in these studies. Both studies were conducted in accordance with the Declaration of Helsinki and participants gave written informed consent prior to participation. The Exercise PRO-MS study was approved by the medical ethics review board (MERB) of the VU University Medical Center (VUmc), Amsterdam. For the gait clinical care cohort, ethics approval was waived by the MERB of the VUmc, Amsterdam. Reference number Exercise PRO-MS (METC 2019.676), Reference number gait clinical care cohort (VUmc 2020.461).

In the current analysis pwMS, age ≥18 years with an EDSS of 2.0–6.0, without contraindications for CPET (i.e. no known cardiovascular, pulmonary or metabolic disease or symptoms of cardiovascular disease) were included; cohort-specific inclusion criteria are described below.

*Exercise PRO-MS study.* The Exercise PRO-MS study is a clinical exercise trial, with 4 measurement sessions each 16 weeks apart (13). PwMS were included in the Exercise PRO-MS study if they had gradual progression of neurological symptoms for 2 years or more. The current study used data from the first or second baseline measurement (i.e. extended baseline period). In most cases data from the first measurement was used, except for 3 participants in whom there was no steady-state oxygen uptake during walking or no maximal CPET. In these three cases, data from the second baseline measurement was used.

*Clinical Care Protocol for Gait Disorders in MS.* As part of usual care at the Department of Rehabilitation Medicine of Amsterdam UMC, location VUmc, pwMS who visit a rehabilitation physician with a question regarding their walking problems are referred for extensive gait analysis and CPET. Only pwMS who are able to walk for at least 6 min without a walking device were eligible. For research purposes, participants gave informed consent for data collection and use.

### Procedures

All participants were scheduled for a CPET and a 6-min overground walking trial within a 2-week period. The median time interval between CPET and walk test was 6 days. Time between measurements was longer than the anticipated 2 weeks in 9 participants, due to sickness or the absence of a participant or assessor, with a maximum gap of 11 weeks in 1 participant.

*Demographics and anthropometrics.* Before the CPET and walking trial, age, sex, MS subtype, disease duration, height, weight and body mass index (BMI) were determined. In addition, a neurological examination was performed to determine disease severity, as measured by the EDSS (14).

*Aerobic capacity.* V̇O_2peak_ and oxygen uptake at VT1 (V̇O_2VT1_) were derived from breath-by-breath gas exchange measurements (Cosmed Quark, Cosmed Benelux BV, The Netherlands) during a CPET on a cycle ergometer (Lode Excalibur Sport, The Netherlands) (15). All participants performed a ramp protocol (5–25 Watt/min) after a 3-min rest phase and a 3-min cycling warm-up phase at 0 Watt. The CPET is the gold standard when determining cardiorespiratory fitness (16), and is considered a valid and safe measure of cardiorespiratory fitness in most pwMS (17).

*Energy demands of walking.* All participants performed a 6-min overground walk test at a self-selected comfortable walking speed (CWS) on a 27- or 30-m oval track. Breath-by-breath V̇O_2_ was measured using a mobile gas exchange measurement system (K5, Cosmed Benelux BV, The Netherlands) (15). Both CWS (i.e. total distance travelled, in m/360 s) and travelled distance were determined. No verbal encouragement was given to patients during the test, in order to prevent conversation disturbing the gas exchange measurement.

### Data analysis

*Aerobic capacity.* The CPET was considered maximal if participants reached a respiratory exchange ratio (RER) of 1.10 in combination with subjective signs of exhaustion (i.e. Borg rating of perceived exertion ≥ 17) (6, 18). In the Cosmed Omnia software, peak aerobic capacity was determined as the highest registered unprocessed binned time averaged V̇O_2_ over at least 20 s at the end ofthe ramp phase (15, 19).

VT1 was determined by a combination of the V-slope method (i.e. V̇O_2_ plotted against V̇CO_2_, with VT1 as the point where V̇CO_2_ exponentially increases) and the ventilation equivalent method (i.e. time plotted against the ventilatory equivalent (VE) of V̇O_2_ and V̇CO_2_, with VT1 as the point where VE/V̇O_2_ starts to increase) (8).

*Energy demands of walking.* In Matlab (version R2021b Mathwork, Natick, MA, USA) energy demand of walking, or so-called energy expenditure of walking (EEw), in this study presented as mL O_2_/kg/min, unless otherwise indicated, was derived from the binned time averaged last 3 min of interpolated (1 Hz) breath-by-breath data (15). To check for valid steady state V̇O_2_,only a minimal change in the V̇O_2_ slope was accepted (slope of linear curve fit < 0.00025 mL/kg/s) (20).

The energy demand of walking can also be expressed per distance travelled, the so-called energy cost of walking (ECw) in mL O_2_/kg/m or in J/kg/m, calculated using the following equations: EEw in J/kg/m according Lusk’s equation (1924) = (15,962 + 5155 * RER * (V̇O_2_ /1000) and ECw = EEw in either O_2_/kg/min or J/kg/min/(walking speed (m/s)) * 60)) (21).

*Relative aerobic load of walking.* The relative aerobic load of walking was determined as the EEw relative to either V̇O_2peak_ and expressed as%V̇O_2peak_, or relative to V̇O_2VT1_ and expressed as %V̇O_2VT1_.

### Statistical analysis

Statistical analysis was performed using STATA 14 statistical software (Statacorp LP, College Station, TX, USA). Normality of data was checked by visual examination of histograms and the Shapiro–Wilk test. Sex-based differences in EEw, aerobic capacity and relative aerobic load were assessed with an independent samples *t*-test, or Mann–Whitney *U* test when assumptions for normality were not met.

Aerobic capacity was compared with reference values to determine whether patients scored worse than age- and sex-matched HC (22). Participants who scored “very poor” (lowest 3%) or “poor” (lowest 3–11%) according to these reference values were classified as having reduced V̇O_2peak_ (22). To compare the energy demand of walking with that of HC, a standard deviation (SD) of 1.5 or more was ranked as an above-average energy demand (21).

As age, sex, disease severity and disease subtype could potentially affect relative aerobic load, a multiple regression analysis was performed, using the enter method to include these factors (6, 7, 12, 22–25).

Both the EEw and ECw are related to walking speed (26, 27). The relationship between EEw and walking speed is best described by a positive linear relationship, whereas the relationship between ECw and walking speed is best described by a U-shaped curve (26, 27). This study also examined these relationships in the study cohort and additionally examined the relationship between relative aerobic load and walking speed using regression analysis.

## RESULTS

### Participant characteristics

In total, 30 pwMS enrolled in the Exercise PRO-MS study and 24 pwMS enrolled in the Clinical Care Protocol for Gait Disorders in MS. Two participants participated in both studies, but were recruited for the Exercise PRO-MS study prior to enrolment in the Clinical Care Protocol for Gait Disorders in MS. In the Exercise PRO-MS study, 2 participants were not able to walk overground for 6 min and were excluded from the analysis. Another participant in the Exercise PRO-MS study was excluded from the analysis due to an invalid steady state 6-min walk test during the first baseline and a measurement error during the second baseline. Four participants were excluded from the CPET. This resulted in a final group of 45 participants (23 from the Exercise PRO-MS study and 22 from Clinical Care Protocol for Gait Disorders in MS).

Demographics, disease-related characteristics and use of assistive devices during walking are reported in Table I. The Exercise PRO-MS study enrolled only patients with secondary progressive MS, resulting in a majority of patients with secondary progressive MS overall (58%).

**Table I:** Participant characteristics

	Total (*n* = 45)	Females (*n* = 32)	Males (*n* = 13)
Age, years (mean (SD))	48.6 (10.1)	48.2 (10.3)	49.6 (9.9)
Multiple sclerosis subtype, *n* (%) Relapsing-remitting multiple sclerosis Secondary progressive multiple sclerosis Primary progressive multiple sclerosis	16 (36) 26 (58) 3 (7)	12 (38) 20 (63) –	4 (31) 6 (46) 3 (23)
Disease duration, years (median (IQR))	15 (9; 20)	17 (11; 23)	11 (3; 17)
Expanded Disability Status Scale, median (IQR)^a^	4 (3.0; 4.5)	4 (2.8; 4.8)	4 (3.5; 4.5)
Body weight, kg, median (IQR)	77 (67; 83)	73 (66; 82)	79 (76; 83)
Body mass index, kg/m^2^, median (IQR)	24.2 (22.6; 27.7)	24.6 (22.1; 28.6)	24.2 (23.7; 26.1)
Walking aid, *n*, (%) None Cane Crutch Walker	39 (87) 3 (7) 2 (4) 1 (2)	26 (81) 3 (9) 2 (6) 1 (3)	13 (100) – – –
Ankle foot orthosis/functional electrostimulation/Foot-up, *n* (%) Yes No	4 (9) 41 (91)	4 (13) 28 (88)	– 13 (100)

^a^Twenty-two subjects, based on neurological testing reported in patient files.

IQR: interquartile range; SD: standard deviation.

### Aerobic capacity

CPET performance, V̇O_2peak_ and V̇O_2VT1_ are shown in Table II. According to the criteria for maximal exercise testing, 33 participants performed a maximal exercise test. VT1 could still be determined in the 12 participants with a submaximal CPET. V̇O_2peak_ was significantly higher in males (2,366 mL/min) than in females (1,638 mL/min), z = –3.02, *p* = 0.003. When corrected for body weight, males had a higher V̇O_2peak_ (28.9 mL/kg/min) than females (24.2 mL/kg/min), z = –1.72, *p* = 0.08. VT1 was higher in males (1,422 mL/min) than in females (1,115 mL/min), z = –2.83, *p* < 0.005, and VT1 adjusted for body weight was also higher in males (18.0 mL/kg/min) compared with females (14.5 mL/kg/min) z = –1.57, *p* = 0.12.

**Table II:** Aerobic capacity and energy demands of walking in 45 persons with multiple sclerosis

	Total (*n* = 45)	Females (*n* = 32)	Males (*n* = 13)
Cardiopulmonary exercise test			
Test performance Maximal Submaximal	33 (73%) 12 (27%)	23 (72%) 9 (28%)	10 (77%) 3 (23%)
V^˙^O_2peak_, mL/kg/min^a^	25.4 (19.6; 29.8)	24.2 (18.7; 29.6)	28.9 (23.9; 33.9)
V^˙^O_2peak_, mL/min^a^	1,842 (1,499; 2,365)	1,638 (1,432; 1,914)	2,366 (1,917; 2,643)
V^˙^O_2VT1_, mL/kg/min	16.5 (13.1; 20.1)	14.5 (12.8; 20.2)	18.0 (15.9; 20.1)
V^˙^O_2VT1_, mL/min	1,242 (1,036; 1,425)	1,115 (983; 1,310)	1,422 (1,272; 1,624)
Overground walking			
Comfortable walking speed, m/s	1.09 (0.97; 1.24)	1.10 (0.97; 1.24])	1.06 (0.97; 1.35)
Energy expenditure of walking, mL/kg/min	14.3 (2.4)	13.9 (2.2)	15.3 (2.6)
Respiratory exchange ratio	0.82 (0.80; 0.85)	0.82 (0.80; 0.87)	0.82 (0.80; 0.83)
%V^˙^O_2peak_^a^	60.0 (12.8)	62.8 (12.7)	53.6 (10.9)
%V^˙^O_2 VT1_	89.1 (19.9)	91.1 (21.0)	84.3 (16.9)
Energy cost of walking, mL/kg/m	0.22 (0.20; 0.24)	0.22 (0.20; 0.24)	0.24 (0.21; 0.25)
Energy cost of walking, J/kg/m	4.44 (4.11; 4.99)	4.41 (3.95; 4.93)	4.76 (4.17; 5.04)

a *n* = 33 (22 females and 10 males).

Data are presented as means (standard deviation; SD) for normally distributed data or medians (interquartile range; IQR) for non-normally distributed data.

%V^˙^O_2peak_: relative aerobic load relative to peak oxygen uptake; %V^˙^O_2VT1_: relative aerobic load relative to oxygen uptake at the first ventilatory threshold; V^˙^O_2peak_: peak oxygen uptake; V^˙^O_2VT1_: oxygen uptake at the first ventilatory threshold.

### Energy demands of walking

The energy expenditure of walking was lower in females (mean 13.9 mL/kg/min) than in males (mean 15.3 mL/kg/min), t (43) = –1.94, *p* = 0.06 (Table II). In 33 participants the relative aerobic load of walking was 60.0% of the maximal V̇O_2peak,_ and was higher in females (mean 62.8%V̇O_2peak_) than in males (mean 53.6%V̇O_2peak_), t (31)=1.99, *p* = 0.06. Compared with the VT1 threshold, the relative aerobic load of walking was 89.1% relative to V̇O_2VT1_ (in 45 participants), and was higher in females (mean 91.1%V̇O_2VT1_) compared with males (84.3%V̇O_2VT1_) (t (43) = 1.03, *p* = 0.31).

### Diagnostic interpretation

Compared with reference values for HC, 9 pwMS had a below-average aerobic capacity, 11 pwMS had an above-average ECw and 6 participants had aberrant ECw and cardiorespiratory fitness (Table IIIA). Ten of the 33 pwMS who performed a maximal CPET had a relative aerobic load > 100%V̇O_2VT1_ (Table IIIB). Of the 45 pwMS included in the study, 14 (31%) walked above their VT1 (> 100%V̇O_2VT1_) (Table IIIC). These findings indicate a high relative aerobic load, with a substantial number of pwMS walking at an energy expenditure above VT1.

**Table III:** Number of participating persons with MS with normal and affected peak oxygen uptake (V^˙^O2peak) and energy cost of walking (ECw) measures

A		Maximal V̇O_2peak_		
		Normal	Below average	Total
ECw	Normal	7	9	16
	Above average	11	6	17
	Total	18	15	33
B		%V^˙^O_2VT1_ (33 pwMS with CPET max)		
		< 100%	≥100%	Total
ECw	Normal	11	5	16
	Above average	12	5	17
	Total	23	10	33
C		%V^˙^O_2VT1_ (total group)		
		< 100%	≥100%	Total
ECw	Normal	12	7	19
	Above average	19	7	26
	Total	31	14	45

Cross tables with normal or increased energy cost of walking and normal or reduced peak aerobic capacity (A), relative aerobic load < VT1 or ≥ VT1 for participants with a maximal cardiopulmonary exercise test (B) and for the total cohort (C).

%V^˙^O_2VT1_: relative aerobic load relative to VT1; V^˙^O_2peak_: peak aerobic capacity; CPET max: maximal cardiopulmonary exercise test; ECw: energy cost of walking; pwMS: people with multiple sclerosis.

### Relationship between relative aerobic load and patient characteristics

A multiple regression analysis was performed to assess the relationship between relative aerobic load and age, sex, severity of MS (EDSS) and MS subtype. Outcomes are presented in Table IV and Fig. S1. Only EDSS added significantly to the model; *p* = 0.006. The overall model explained 46% of the variance in %V̇O_2peak_ (R^2^ = 0.460).

**Table IV:** Multiple regression analysis of the relative aerobic load of walking by age, sex, disease severity and multiple sclerosis (MS) subtype

	%V^˙^O_2peak_					%V^˙^O_2VT1_				
	B	SE	95% CI		*p*-value	B	SE	95% CI		*p*-value
			Lower	Upper				Lower	Upper	
Intercept	27.6	9.8	7.5	47.7	0.009	60.3	17.9	24.0	96.5	0.002
Age (years)	0.3	0.2	–0.1	0.7	0.116	0.5	0.3	–0.2	1.2	0.155
Disease Severity (EDSS)	6.4	2.1	2.0	10.7	0.006	1.4	3.3	–5.2	8.0	0.679
Male sex (Reference cat. Female)	–7.7	4.5	–16.9	1.5	0.098	–4.7	7.3	–19.4	10.0	0.522
MS-Subtype: SPMS (Reference cat. RRMS)	–6.5	5.7	–18.2	5.3	0.267	2.6	8.3	–14.1	19.3	0.756
MS-subtype: PPMS (Reference cat. RRMS)	–7.4	8.9	–25.6	10.9	0.416	–9.8	15.2	–40.5	20.9	0.523

%V^˙^O_2peak_: relative aerobic load relative to peak oxygen uptake; %V^˙^O_2VT1_: relative aerobic load relative to the first ventilatory threshold; 95% CI: 95% confidence interval; B: Beta; cat: category; EDSS: Expanded Disability Status Scale; MS: multiple sclerosis; PPMS: primary progressive multiple sclerosis; RRMS: relapsing-remitting multiple sclerosis; SE: standard error; SPMS: secondary progressive multiple sclerosis.

The association between %V̇O_2VT1_ and age, sex, disease severity and MS subtype was not significant F(5, 39)= 1.12, *p* = 0.36, R^2^ = 0.126, and none of the variables added significantly to the model.

### Relative aerobic load in relation to other physiological measures of walking

**Fig. 1:**
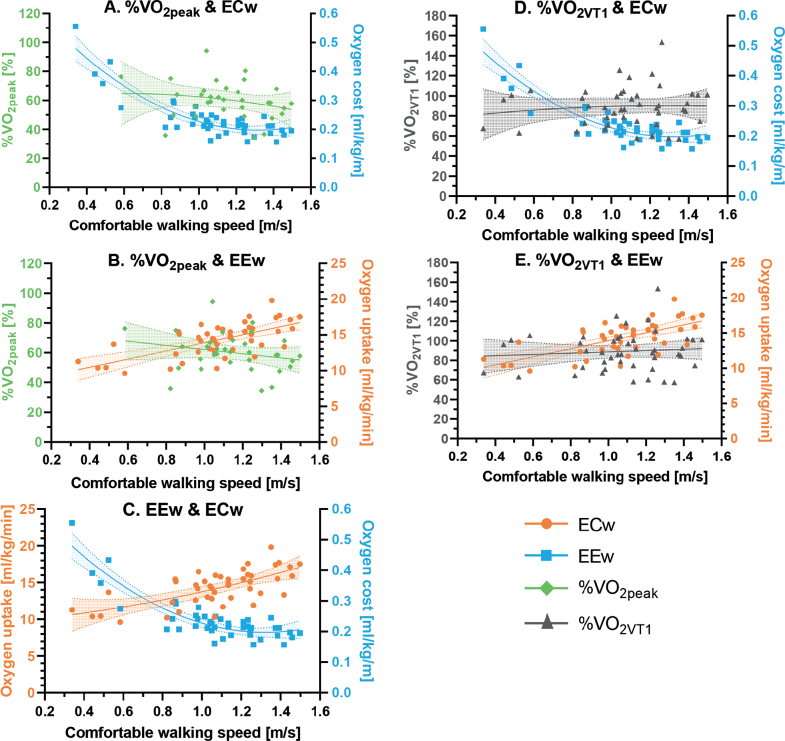
The relationships between comfortable walking speed with: (A) energy cost of walking (ECw) and relative aerobic load relative to peak oxygen uptake (%VO_2peak_), (B) with energy expenditure of walking (EEw) and %VO_2peak_, (C) with EEw and ECw, (D) with ECw and relative aerobic load relative to the first ventilatory threshold (%VO_2VT1_) and, (E) with EEw and %VO_2VT1_.

The current study also investigated the relationship between CWS and EEw per min, ECw per m, and relative aerobic load (Table V and Fig. 1). The relationship between CWS and ECw (mL/kg/m) was best described by a quadratic or U-curve function. The relationship between CWS and EEw (mL/kg/min) was best described by a linear model, with a significant increase in EEw of 5.7ml O_2_/kg/min (95% confidence interval (95% CI) 3.72; 7.67) for a 1 m/s faster walking speed. However, the slope for relative aerobic load was non-significant for both %V̇O_2peak_ –13.9 (–35.3; 7.5) and %V̇O_2VT1_ 6.5 (–15.6; 28.7).

**Table V:** Relationship between comfortable walking speed and energy demands of walking in persons with multiple sclerosis (pwMS)

		B	SE	95% CI		*p*-value
				Lower	Upper	
Energy cost of walking	Intercept	0.71	0.05	0.61	0.80	< 0.001
	CWS	–0.77	0.10	–0.98	–0.57	< 0.001
	CWS^2^	0.29	0.05	0.19	0.40	< 0.001
Energy expenditure of walking	Intercept	8.20	1.08	6.01	10.38	< 0.001
	CWS	5.70	0.98	3.72	7.67	< 0.001
%VO_2peak_	Intercept	76.02	12.24	51.06	100.98	< 0.001
	CWS	–13.93	10.49	–35.32	7.47	0.194
%VO_2VT1_	Intercept	82.14	12.15	57.63	106.65	< 0.001
	CWS	6.51	11.00	–16.67	28.69	0.557

95% CI: 95% confidence interval; %V^˙^O_2peak_: relative aerobic load relative to peak aerobic capacity; %V^˙^O_2VT1_: relative aerobic load relative to the first ventilatory threshold; CWS: comfortable walking speed; SE: standard error.

## DISCUSSION

The aim of this study was a better understanding of the relative aerobic load of walking in pwMS, based on aerobic capacity and the energy expenditure of walking. The study found a mean relative aerobic load of walking (at CWS) of 60.0%V̇O_2peak_ and 89.1%V̇O_2VT1_. The study also found that almost one-third of pwMS walked above their VT1 during CWS, indicating a potentially unsustainable relative aerobic load.

### Relative aerobic load of walking

To date, the relative aerobic load of walking has only been assessed in relation to amputation (11), stroke (10), cerebral palsy (28, 29) and matched HC in these studies. The relative aerobic load of walking in HC in these studies varied between 36.2%V̇O_2peak_ (10) and 48.7%V̇O_2peak_ (11), which is substantially lower than 60.0%V̇O_2peak_ in our MS population. The relative aerobic load in traumatic amputation, stroke and cerebral palsy patients was approximately 50%V̇O_2peak_, and was even higher (73%V̇O_2peak_) in patients with vascular amputation (10, 11, 29). In the only study that investigated %V̇O_2VT1_, the HC group walked at 65%V̇O_2VT1_ (10), while a chronic stroke population allocated to more and less impaired groups had relative aerobic loads of 102%V̇O_2VT1_ and 97%V̇O_2VT1_, respectively. The relative aerobic load of 89.1%V̇O_2VT1_ in pwMS in the current study is therefore higher relative to HC but lower than chronic stroke patients.

Relative load might substantially influence walking. Approximately one-third of the current MS study population walked above their VT1, which will lead to exertion during comfortable walking. Earlier literature has shown that using ≥ 50% of V̇O_2peak_ during the day results in exertion (30). The current study found %V̇O_2peak_ of walking to be 60% at CWS, not taking into account factors in daily living, such as uneven surfaces or dual tasking. These additional factors are expected to contribute to an even higher relative aerobic load and will probably affect daily functioning, societal participation and quality of life.

### Aerobic capacity

An important component of relative aerobic load is aerobic capacity. A median V̇O_2peak_ of 25.4 mL/kg/min (IQR 19.6; 29.8) is in line with review findings in pwMS (mean V̇O_2peak_ 25.5 mL/kg/min, SD 5.2), and is lower compared with HC (mean V̇O_2peak_ 30.9, SD 5.4) (6). However, comparing V̇O_2peak_ with reference values for age- and sex-matched HC is difficult, as several reference values exist and, due to heterogeneity in study populations and methodologies, an ideal set of reference values is not yet available (25). In the current study we compared our data with normative values used in clinical practice, derived from a literature review that included V̇O_2peak_ data from 141 healthy, untrained, sedentary or mildly active individuals aged 6–75 years, compiled from 62 different studies conducted in North America, Europe, and Israel, all published prior to 1986. The testing modalities consisted of cycle ergometer (32 studies), treadmill (25 studies) or recumbent stepper (5 studies) (22). Using these normative values, 15 people were classified as having reduced V̇O_2peak_ (below the 11th percentile), while 18 people had a “normal” V̇O_2peak_ (22). However, other reference values result in different numbers. Compared with Danish norm values 23 people had a V̇O_2peak_ below the 20th percentile (31), compared with German norm values 16 people scored below the 20th percentile (32), and compared with reference values from the USA only 9 participants had a V̇O_2peak_ below the 20th percentile (33). On average, this is in line with the primarily used reference values in this study.

The ventilatory threshold VT1 marks the point at which the anaerobic energy supply becomes predominant, resulting in physiological changes that will lead to unsustainability of the activity (34). VT1 has received little attention, especially in pwMS (35, 36). Data from a large cohort of healthy individuals (N = 8,155) in the USA found a VT1 of 14.9 mL/kg/min (SD 4.1), with a significant difference between males (mean 15.3 mL/kg/min, SD 3.9) and females (mean 13.3 mL/kg/min, SD 4.3) (37). This is lower compared with our finding of 16.5 mL/kg/min for the total group, with 18.0 mL/kg/min in males and 14.6 mL/kg/min in females. Previous studies in pwMS reported V̇O_2VT1_ levels ranging from 13.6 to 18.7 mL/kg/min (35, 36), similar to the current findings. The higher V̇O_2VT1_ found in pwMS might be related to increased energy expenditure during daily activities, which might inflict training effects already during these activities (38). The VT1 could be an important measure, since this threshold is a marker for exhaustion during prolonged activities, which, in turn, impacts specific daily activities, such as walking (34). However, as determination of VT1 is rater-dependent and several different measurement techniques have been described (i.e. V-slope method, equivalents method), VT1 may be more prone to error (39). It is also questionable whether a ramp protocol is the best approach to determine VT1, as increments are very short (i.e. seconds), whereas increments of several minutes might allow more precise determination of VT1 (40, 41). An appropriate CPET protocol and set of reference values for both V̇O_2peak_ and V̇O_2VT1_ are very important in the valid interpretation of these measures of aerobic fitness.

### Energy demands of walking

When determining the relative aerobic load of walking, the other important component is energy expenditure during walking. According to Ainsworth’s compendium of physical activity in healthy people, walking at 1.1 m/s will result in an energy expenditure of 10.5 mL/kg/min (42), which is lower than the 14.6 mL/kg/min found in the current study. However, the energy expenditure per min of walking depends heavily on walking speed. In line with previous research, in the current study higher walking speeds resulted in significantly higher energy expenditure (26). To correct for dependency on walking speed ECw is often presented (27), expressed as mL O_2_/kg/m or J/kg/m. The ECw of participants in the current study was higher (median 4.4 J/kg/m) compared with HC aged 18–41 years (mean 3.4 J/kg/m, SD 0.4) and adults > 59 years (mean 3.8 J/kg/m, SD 0.4) (21). A recent systematic review reported an ECw of 0.18 mL O_2_/kg/m in HC and 0.23 mL O_2_/kg/m in pwMS, the latter being in line with the 0.22 mL O_2_/kg/m found in the current study (43).

The relationship between walking speed and ECw can be described by a U-shaped curve, which was also the case in the current study. Habitual walking speed is assumed to optimize to the lowest ECw (27). PwMS, however, walk at a lower CWS compared with HC (23), which might explain the increased ECw in this group (43). Nonetheless, in gait laboratory experiments pwMS are able to increase their walking speed to, for example, 1.5–1.8 m/s, which reduced or maintained the ECw (43). Clearly, the relationship between CWS and ECw is less well understood in pwMS. The current study was not able to demonstrate a significant relationship between the relative aerobic load of walking and walking speed, which may indicate that pwMS might chose a walking speed of approximately 60%V̇O_2peak_ or just below VT1, as higher loads could be unsustainable for prolonged walking.

### Strengths and limitations

This is the first study to examine the relative aerobic load of walking in pwMS who experience walking problems during daily living. One limitation is that V̇O_2peak_ andV̇O_2VT1_ were measured during cycle ergometer CPET and compared with the energy expenditure of walking. Thus, relative aerobic load was a product of both cycling and walking assessments. In the current study, treadmill tests were not deemed feasible for pwMS with walking impairments; hence cycle ergometer CPETs were chosen despite possible deviation in relative aerobic load.

### Clinical implications and future research

The relative aerobic load of walking is a comparatively new and unexplored measure of walking, which could potentially improve the clinical understanding of walking problems experienced by pwMS. It is often assumed that people adjust their walking speed to minimize the ECw (27, 43). However, pwMS with a reduced aerobic capacity might reduce their walking speed to lower energy expenditure and relative aerobic load, subsequently walking at a high energy cost. Optimizing towards an optimal ECw in these patients may result in a high, unsustainable aerobic load relative to V̇O_2peak_ orV̇O_2VT1_.

To better understand and interpret a person’s relative aerobic load of walking, it would be useful to examine the relationship with different walking speeds in the same person, and establish an appropriate set of reference values based on a large group of both patients and HC.

The relative aerobic load of walking has important clinical implications, not only for walking but also for other daily activities. As both a reduced aerobic capacity and an increased energy demand contribute to a high relative aerobic load, interventions should take these contributing factors into consideration. When a high relative aerobic load is caused by a reduction in V̇O_2peak_ or VT1, exercise interventions to improve cardiorespiratory fitness might be warranted (6). A meta-analysis demonstrated that aerobic exercise interventions improve aerobic capacity in pwMS (6). To specifically improve VT1, training intensities near or above VT1 might result in better outcomes (38, 44). Gait training, unsupported or supported, can help alleviate peripheral mitochondrial dysfunction and decrease additional anaerobic energy supply (45). A reduction in energy expenditure during walking can be effectively achieved with assistive devices, such as an ankle-foot orthosis or push-off specific muscle strength training (46, 47).

In conclusion, the relative aerobic load of walking in pwMS is high, and close toV̇O_2VT1_. To walk at a sustainable energy expenditure relative to either V̇O_2peak_ orV̇O_2VT1_, pwMS may reduce their CWS, resulting in an increased ECw per metre. The relative aerobic load of walking can differentiate between problems related to aerobic capacity and those related to gait, and is therefore a valuable measure in clinical rehabilitation.

## Supplementary Material

RELATIVE AEROBIC LOAD OF WALKING IN PEOPLE WITH MULTIPLE SCLEROSIS

## Trial registration

The Exercise PRO-MS study was registered prospectively at the Dutch Trial Registry (number NL8265, date 06-01-2020). The Clinical Care Protocol for Gait Disorders in MS was not prospectively registered.
