# Supplementary figures and images for: RELATIVE AEROBIC LOAD OF WALKING IN PEOPLE WITH MULTIPLE SCLEROSIS
Source: J Rehabil Med. 2024 Feb 14;56:13352. doi: 10.2340/jrm.v56.13352 (PMC10875758; doi:10.2340/jrm.v56.13352)

Fig. S1. Determinants of % $\dot{V}O_{2peak}$  and %  $\dot{V}O_{2VT1}$

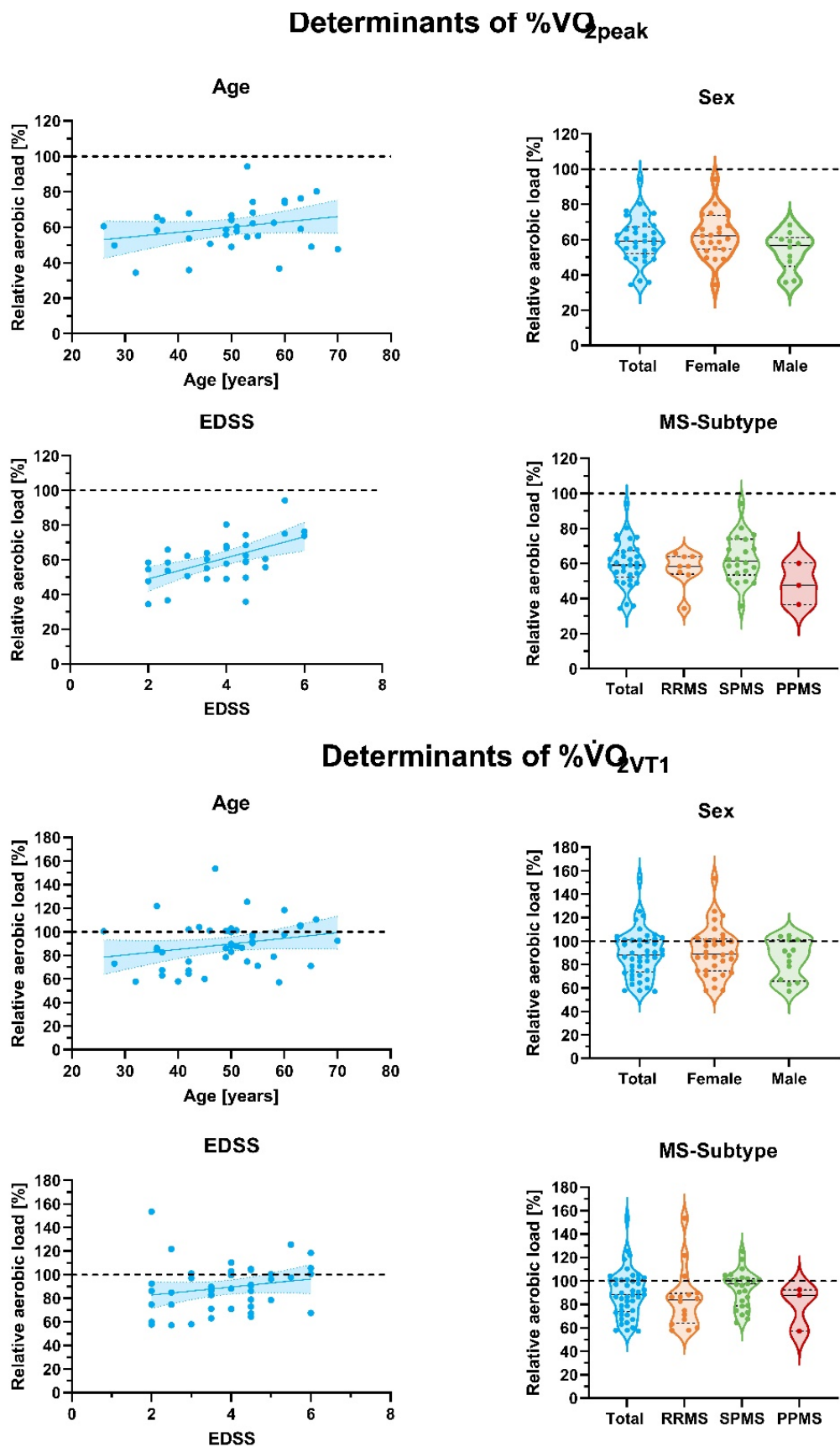

Supplement: RELATIVE AEROBIC LOAD OF WALKING IN PEOPLE WITH MULTIPLE SCLEROSIS [file JRM-56-13352-s1.pdf]
